# Quantitative evaluation of the drivers of species richness in a Mediterranean ecosystem (Cape, South Africa)

**DOI:** 10.1093/aob/mcad134

**Published:** 2023-09-15

**Authors:** Michael D Cramer, G Anthony Verboom

**Affiliations:** Department of Biological Sciences, University of Cape Town, Rondebosch, Cape Town, South Africa; Department of Biological Sciences, University of Cape Town, Rondebosch, Cape Town, South Africa

**Keywords:** Biodiversity, biotic feedback, many individuals hypothesis, niche, nutrient scarcity, spatial heterogeneity, species richness, temporal heterogeneity, water–energy hypothesis

## Abstract

**Background and Aims:**

Mediterranean ecosystems have a high vascular plant species richness (SR) relative to their surface area. This SR, representing the balance between speciation and extinction, has been attributed to multiple mechanisms that result in both high rates of speciation and/or low rates of extinction. An abiding question is, however, what is special about Mediterranean ecosystems that enables this high SR? Apart from the long-term climatic stability of the region, SR has also been related to resource availability, the many individuals hypothesis, resource spatial heterogeneity, temporal heterogeneity and biotic feedbacks.

**Methods:**

Spatial patterns of species richness were related to climatic, edaphic and biotic variables and to spatial variability within the Greater Cape Floristic Region (GCFR) of South Africa. Boosted regression tree models were used to explore the strength of relationships between SR and environmental predictors related to each hypothesized mechanism.

**Key Results:**

Water availability (i.e. precipitation) was a stronger predictor of SR than potential evapotranspiration or temperature. Scarcity of nutrients was also related to SR. There was no indication that SR was related to the density of individuals and only temporal heterogeneity induced by fire was related to SR. Spatial heterogeneities of climatic, edaphic and biotic variables were strongly associated with SR. Biotic interactions remain difficult to assess, although we have some evidence for a putative role in regulating SR.

**Conclusions:**

While the lack of ecosystem-resetting disturbances (e.g. glaciation) is undoubtedly a key requirement for high species accumulation, predictably, no one explanation holds the key to understanding SR. In the GCFR high SR is the product of a combination of adequate water, nutrient scarcity, spatial and temporal heterogeneity, and possibly biotic feedbacks.

## INTRODUCTION

Darwin recognized the potential of the lapse of geological time as a contributor to regional species richness (SR; Hopper and Lambers, 2009). Together with appreciation of the role of ecosystem-resetting disturbances such as tectonic uplift and glaciation (Kreft and Jetz, 2007), this is a major underpinning of our understanding of latitudinal gradients in SR. Co-variation of other factors may, however, partially obscure the simple truth that the accumulation of species requires time. These other factors include the association between climate and SR that has been encapsulated in the ‘water–energy dynamics hypothesis’ (Hawkins *et al*, 2003), the association of SR patterns with aspects of environmental heterogeneity that drive regional species turnover (Gaston, 2000), the role of soils in promoting SR (e.g. Güsewell 2004) and the role of biotic interactions as a driver of SR (Schemske, 2009). Mediterranean-type ecosystems are home to some of the highest levels of SR outside the tropics (Rundell *et al.*, 2016). These ecosystems have not recently (in evolutionary time) experienced ecosystem-resetting disturbances due to their temperate latitudes and oceanically buffered climates. The importance of age and climatic stability for SR has been captured by the monicker ‘old climatically buffered infertile landscapes’ (OCBILs) juxtaposed with ‘young often disturbed fertile landscapes’ (YODFELs, Hopper, 2009). As OCBILs, these systems have had time to accumulate species, but other drivers of SR may include rates of diversification/extinction (e.g. Hopper *et al.*, 2009; Verboom *et al.*, 2009), climate (Thuiller *et al.*, 2006), environmental heterogeneity (Cramer and Verboom, 2017), the presence of glacial refugia (Verboom *et al.*, 2009), nutrient-impoverished soils (Hayes *et al*., 2021) and biotic interactions (Mod *et al.*, 2015).

While the lack of ecosystem-resetting disturbances contributes to the age of Mediterranean ecosystems, this also contributes to the occurrence of old highly leached soils that is exacerbated by both nutrient-poor underlying geologies and the possibility that Mediterranean winter-rainfall contributes to leaching through low evapotranspiration in the rainy season (Cramer and Hoffman, 2015). Both positive and negative effects of soil nutrients on SR may be anticipated. For example, Cornwell and Grubb (2003) found hump-shaped patterns of SR with soil nutrients in central Europe that differed between vegetation types. Despite this, Huston (1980) found a negative relationship between soil fertility and SR of Costa Rican forests. Indeed, the scarcity of phosphorus (P) has driven multiple adaptations of plants to survive in infertile landscapes (Hopper *et al.*, 2021). For example, SR increases with declining soil P, especially for Proteaceae, resulting in the suggestion of faster diversification in lineages adapted to P-impoverished soils (Hayes *et al.*, 2021). The key innovations thought to facilitate this diversification are functional cluster roots, low leaf P concentrations and high seed P concentrations. Güsewell (2004) suggested that numerous chemical forms of P and the low diffusional mobility of P in soil might contribute to limiting interspecific competition for P allowing greater species coexistence. Indeed, plants with diverse adaptations to low-P soils (e.g. cluster roots) co-occur with non-cluster rooted mycorrhizal plants. This has been explained as being due to facilitation between cluster rooted species and non-cluster rooted species (Hopper *et al.*, 2021), but it is also possible that a diversity of trait-assemblages (Douma *et al.*, 2012) work equally well in a given habitat. For example, slow growth and small stature might compensate for the lack of P-acquiring cluster roots. Consequently, although there might be an expectation of a hump-shaped pattern of SR with soil nutrients, the hump in SR is likely to be displaced towards lower soil nutrients.

Spatial environmental heterogeneity promotes SR both by facilitating coexistence of species at landscape or regional scales (Wright, 1983; Kerr and Packer, 1997; Silvertown, 2004; Gillman and Wright, 2014), and by promoting species diversification. Environmental heterogeneity plays a central role in speciation both through its provision of resource gradients along which adaptive divergence can occur and by providing barriers to gene flow (Wiens, 2004; Dynesius and Jansson, 2014; Ellis *et al.*, 2014). While global models of SR have been remarkably successful in predicting SR (Kreft and Jetz, 2007), the failure to successfully model the biodiversity of the Cape Floristic Region (CFR) and the South West Australian Floristic Region (SWAFR) may be due to insufficient consideration of the heterogeneity of these areas (Cramer and Verboom, 2017). This heterogeneity operates at different scales in the CFR and SWAFR, with the former having greater topographic variability and finer scale turnover (van Mazijk *et al.*, 2021). For the SWAFR, the turnover is associated with edaphic turnover between sediments deposited at different times. Spatial heterogeneity is thus an important component of explaining Mediterranean ecosystem SR. Furthermore, the degree of spatial heterogeneity of resources may change with resource levels. For example, in South Africa temperature spatial heterogeneity decreases with mean temperature, whereas water availability (i.e. precipitation – potential evapotranspiration) spatial heterogeneity increases with its mean value (Cramer and Verboom, 2017).

Seasonal variability and fires provide two sources of temporal heterogeneity. This variability in environmental conditions allows the coexistence of species with varying competitive abilities in what has been termed ‘the storage effect’ (Warner and Chesson, 1985; Yeaton and Bond, 1991, Chesson, 2000). Seasonal variation in resources is perhaps most relevant for annual species, since perennial species need to cope with the full annual cycle and may be able to invest in structures that enable survival in challenging times and are able to delay reproduction (Lundgren and Des Marais, 2020). Annuals are relatively rare in the more infertile landscapes of the Greater Cape Floristic Region (GCFR; Born *et al.*, 2007) because they require ready access to nutrients to fund rapid growth (Verboom *et al.*, 2012) restricting their distributions to the more fertile portions of the landscape. The potential role of seasonal variability in contributing to SR is therefore not clear. Fire is common in open-canopy ecosystems and has been an important evolutionary driver in Mediterranean ecosystems (He *et al.*, 2016) causing changes in species composition in the post-fire environment (Thuiller *et al.*, 2007). In the GCFR, for example, regular fires in open-canopy fynbos do not commonly penetrate into Afromontane forests (Coetsee *et al.*, 2015) creating the opportunity for spatial environmental heterogeneity. Variability in fire intensity also contributes to SR through temporal and spatial heterogeneity (Song and Liu, 2019). Fires not only reset the ecological landscape allowing seeds to germinate (e.g. Hanley *et al.*, 2003) but also release a post-fire nutrient flush, both of which contribute to temporal heterogeneity. For example, in the GCFR, post-fire legumes thrive for a few years, but are not a dominant component of the mature fynbos (Cocks and Stock, 2001; Power *et al.*, 2010). The post-fire nutrient flush has another consequence in that repeated fires lead to the loss of nutrients over time because ecosystems are leaky (Pellegrini *et al.*, 2015). Nutrients may be lost as wind-blown ash or from runoff or leaching into water bodies. Fire may therefore contribute to SR by creating spatial and temporal heterogeneity and by driving specializations to fire.

Specializations of fynbos to fire, whether exaptations or adaptations (Bradshaw *et al.* 2011), include whole-plant fire responses (e.g. resprouting vs. reseeding), serotiny, and fire-stimulated seed germination and flowering (Keeley *et al.*, 2011; Lamont *et al.*, 2020). While open-canopy vegetation may be more prone to fire, fires also maintain open vegetation. The time between recurrent fires sets a limit on the size of plants and requires that plants set seed within the window of the fire return interval. In addition to the limitation imposed by fire on growth time, fire also reduces nutrient availability creating a constraint on plant growth (Lenton, 2001). Low-stature vegetation consisting of smaller individuals may allow the coexistence of more species since there could be more individuals in a given area (Aarssen *et al.*, 2006). These authors suggest that the abundance of smaller plant species is due to their higher likelihood of leaving descendants through higher fecundity compared to larger species, leading to greater speciation opportunities and lower extinction rates for smaller species. This may relate to the more individuals hypothesis (MIH; Storch *et al.*, 2018) in which the total number of individuals limits the number of species that can have viable populations in that environment (Gaston, 2000). This MIH hypothesis has been invoked to explain the latitudinal gradient in SR and is perhaps an explanation for the latitudinal ‘water–energy’ relationship (Hawkins *et al.*, 2003) in which more resource allows more individuals and therefore more species (Evans *et al.*, 2005). Evidence for the MIH operating for vascular plants is, however, limited globally (Storch *et al.*, 2018). In the context of South Africa there is also a lack of evidence for ‘energy’ relationships with SR, although water is weakly positively related to SR (Hoffman *et al.*, 1994; Cramer and Verboom, 2017). Indeed, infertile fire-prone landscapes are likely to cause a break-down in water–energy relationships because water and energy may not be the limiting factors on plant size in these environments. Nutrient-poor soils enforce slow growth and open-canopy vegetation that is susceptible to disturbances (e.g. fire and herbivory). This could favour low-stature vegetation with more individuals and high SR. Based on the high SR of bacteria and fungi, it is incontrovertible that small size at least creates the opportunity for high SR (O’Mallet and Dupré, 2007).

Despite early recognition of their potential contribution to SR, biotic interactions have enjoyed relatively little attention as a driver of SR (Schemske, 2009). Several authors (e.g. Dobzhansky, 1950; Fischer, 1960) have suggested, however, that biotic interactions are more important as contributors to SR in the tropics, whereas abiotic factors are more important outside that zone. Schemske *et al.* (2009) reviewed the evidence for biotic interactions and traits related to biotic interactions. Examples include the fact that more plants have larger more faunally dispersed seeds in the tropics (Moles *et al.*, 2007) and that the incidence of fungal endophytic infections increases from 1 % in the Arctic to 99 % in the tropics (Arnold and Lutzoni, 2007). Over a diversity of organisms and traits, Schemske *et al.* (2009) concluded that biotic interactions are frequently more important as determinants of SR in tropical regions. These biotic interactions effectively multiply niche space because of the complexity of interactions between all interacting taxa. In species-rich ecosystems such as Mediterranean ecosystems, the opportunity for such interactions may be increased, justifying the suggestion of the importance of biotic interactions (e.g. competition and facilitation) in determining SR (e.g. Cowling *et al*., 1996).

Pianka (1966) suggested that compounding the hypotheses for the latitudinal gradient of SR may result in untestable theory. The rates of speciation and extinction are, however, influenced by many factors and we should resist the appeal of Occam’s razor in down-playing multi-factor explanations for SR (Hopper *et al.*, 2021). We thus need to consider a diversity of causal predictors of SR. The question is as to what the minimum set of causal predictors of SR might be. For example, several authors have included topography in models of SR and these models do perform well in predicting SR (Thuiller *et al.*, 2006; Kreft and Jetz, 2007). However, plants do not experience topography as a resource and the reason that topography works so well is that it captures the variability of edaphic conditions, temperature, radiation and precipitation (Moeslund *et al.*, 2013). In addition, topographic heterogeneity may result in community isolation and provide opportunity for speciation in isolation and for the existence of refugia (Verboom *et al.*, 2003). Thus, if we want to understand the drivers of SR, rather than just predict it, using the variables that relate to the resources that plants actually use is important (Cramer and Verboom, 2017).

We aimed to evaluate ecological explanations of GCFR SR rather than expressly historical explanations, but accepting that the high SR of the GCFR requires relatively long periods of climate stability without ecosystem-resetting disturbances (e.g. glaciation). Our aim was to provide a quantitative assessment of some alternative explanations for SR within the region. We hypothesized that SR (1) requires both spatial and temporal abiotic and biotic heterogeneity; (2) is higher in areas with more resources and more individuals; (3) conversely, is increased in areas of nutrient impoverishment; and (4) may be increased by biotic feedbacks. Using species records from across the GCFR in South Africa we compared the degree of SR with measures of ecosystem resources and resource heterogeneity (climatic and edaphic), variability in the fire regime, and the extent of bare ground and the number of individuals.

## METHODS

### Plant SR data

A herbarium-based database of vascular plant (VP) species for South Africa was compiled previously, based on the GBIF database (Global Biodiversity Information Facility; www.gbif.org) from which alien species were removed and names were reconciled (Cramer and Verboom, 2017). These data are sometimes associated with GPS coordinates, but commonly (i.e. older collections) are specified per quarter degree square (QDS). We adopted the QDS as our unit of analysis and expressed SR on a per area basis (e.g. QDS area ~670 km^2^). We omitted QDSs that intersected the coastline since there is uncertainty about the SR of these (smaller) terrestrial areas. These GBIF-derived data have the problem that they may be subject to collection bias. Previously we (Cramer and Verboom, 2017; Van Mazijk *et al.*, 2021) argued that uncorrected data (i.e. not subject to rarefaction analysis) were more suitable for estimating SR and showed that these data were consistent with other independent sources. The GCFR was our area of interest comprising the area occupied by the Succulent Karoo and Fynbos Biomes (Mucina and Rutherford, 2006) but bordering the Nama Karoo and Desert biomes. The 2012 National Vegetation Map produced by the South African National Biodiversity Institute (http://bgis.sanbi.org) was used to identify the biomes into which >50 % of each QDS fell. The Fynbos biome includes fynbos, strandveld, renosterveld and Afromontane forests which might deserve to be treated as separate floristic units (Bergh *et al.*, 2014). The coarse resolution of QDS does not allow analysis of these smaller fragments.

### Environmental data

Wind and solar radiation data were obtained from the Worldclim2 (https://worldclim.org/) database (Fick and Hijmans, 2017). These data are averaged from the years 1970–2000, and are interpolated between weather stations to ~1-km^2^ resolution based on longitude, latitude, elevation, distance to coast and cloud cover. They are based on regional models rather than a single global model, providing more accurate climate estimates than the original Worldclim dataset (Hijmans, 2015). The bioclimatic variables (bio1–bio19) were extracted from Climatologies at High resolution for the Earth’s Land Surface Areas (CHELSA, accessed 2023, https://chelsa-climate.org; Karger *et al.*, 2017). The bioclimatic variables are derived from monthly maximum, mean precipitation and mean temperature values. These variables include: mean annual temperature (MAT), mean diurnal range (mean of monthly range, i.e. max temp − min temp), isothermality, temperature seasonality (standard deviation), max temperature of warmest month, min temperature of coldest month, temperature annual range, mean temperature of wettest quarter, mean temperature of driest quarter, mean temperature of warmest quarter, mean temperature of coldest quarter, mean annual precipitation (MAP), precipitation of wettest month, precipitation of driest month, precipitation seasonality (coefficient of variation), precipitation of wettest quarter, precipitation of driest quarter, precipitation of warmest quarter and precipitation of coldest quarter.

The soil data for this study were from regionally modelled soil characteristics in the GCFR of South Africa (Cramer *et al.*, 2019). These included pH (KCl extract), electrical conductivity (EC, mS m^-1^), total N (%, w/w), total C (%, w/w), extractable P (mg kg^−1^), extractable K (cmol^+^ kg^−1^) and extractable Na (cmol^+^ kg^−1^). These regional data are more representative of the edaphic properties of the region than the current global Soilgrids product (Hengl *et al.*, 2017), but nevertheless represent a spatial interpolation.

While potential evapotranspiration (PET) is related to energy inputs (temperature, solar radiation, wind) and precipitation to water inputs, the utilization of these resources is probably constrained by their seasonal variations and the availability of nutrients. The normalized difference vegetation index (NDVI), representing the amount and greenness of the foliage, may serve as a better proxy for available resources since this integrates the availability of water, light, temperature and nutrients. Average annual NDVI was calculated at 250-m resolution using the 10-d maximum-value composite procedure for the period 2001–2010 (Swets *et al.*, 1999). The data were retrieved from eMODIS TERRA (US Geological Survey Earth Resources Observation and Science Center), which corrects for atmospheric effects such as molecular scattering, ozone absorption and aerosols. Interpretation of NDVI as a proxy for ‘energy’ in driving SR is, however, complicated by the fact that NDVI relates to vegetation density, which could independently influence SR (e.g. through greater light–niche segregation potential).

Fire frequency is related to temporal heterogeneity in that fires are a disturbance that resets several ecosystem properties (e.g. competitive dynamics, species composition and nutrient status). Fire frequency data were derived from the monthly MODIS burned area product (MODIS Burned Area Product 5.1 at 250-m, accessed 2022) from the period 2000–2021. The BurnDate field of these data was used to count the number of fires over the period of observation for each pixel. While fires may be anthropogenically induced and suppressed, the flammability of vegetation is strongly determined by the vegetation characteristics (van Wilgen *et al.*, 2010). Thus, although fire is not a biotic variable, vegetation flammability does partially determine the frequency and intensity of fire.

Topographical elevation (DEM) data were derived from the Shuttle Radar Topography Mission (SRTM, accessed 2019, https://earthexplorer.usgs.gov.) at 30-m horizontal and 16-m vertical resolution. Every eight 30-m^2^ pixels of this dynamic elevation model were first aggregated to a 240-m horizontal resolution and used to produce the aspect (recoded to eight cardinal directions as a factor), landscape slope and ruggedness (topographic heterogeneity) using the *terrain* function in the raster library (Hijmans, 2022) in R (R Core Team, 2022). All environmental data were finally aggregated to 1-km^2^ resolution based on the GCFR soil data using bilinear and nearest neighbour methods for continuous and categorical data, respectively, using *resample* in raster in R.

The Global Ecosystem Dynamics Investigation Lidar data (GEDI L2A Raster Canopy Top Height, Version 2) is a point-based measure with a spatial resolution of 25 m and a vertical resolution of 1 m. A raster version of these data (GEDI02_A_002_MONTHLY) was obtained from Google Earth Engine (Gorelick *et al.*, 2017). From this we extracted the bands for relative height metrics at 98 % (i.e. close to the top of the vegetation) and the per cent non-vegetated and per cent tree cover from MODIS MOD44B V6 data. Within the Fynbos biome, heights exceeding 3 m are unusual. The vegetation height of this biome may be influenced by the occurrence of Afromontane forests in the quarter degree grids that are otherwise dominated by Fynbos, but also the possible occurrence of alien invasions (commonly *Pinus* species; Holmes *et al.*, 2020). To restrict the influence of alien species on these metrics we excluded all ‘transformed’ land from consideration using the South African Landcover map (30-m resolution), but this exclusion would be better realized with a high-resolution alien invasion map, which is unfortunately not available. The landcover classes in the South African National landcover 2018 product (DEA E1434 Land-Cover; www.environment.gov.za) were categorized as transformed vs. untransformed using the raster *reclassify* function in the raster library in R (Hijmans, 2022) with the reclassification matrix defined by 0–14 = 1; 21–31 = 1; 14–21 = 0; 31–Inf = 0, and this was used to *mask* the GEDI point data using the raster library to exclude points in transformed areas. The retained individual points were averaged for each QDS. An index for the number of individuals was derived from the per cent cover divided by the relative height, based on the logic that a large proportion of cover for low stature suggests many individuals whereas a similar cover with taller vegetation suggests fewer individuals.

### Data analysis

All analyses were conducted in R (R Core Team, 2022). For each environmental variable we calculated the standard deviation and a spatial coefficient of variation (CV) from the standard deviation divided by the mean of the values within a QDS. Heteroskedasticity is commonly a product of values being less variable as their mean approaches zero (Supplementary Data Fig. S1). Owing to strong correlation between the measured values and their variances, inclusion of both in predictive models is not possible. An alternative expression of the variance is the CV, which also varies with the resource level, but in a less predictable manner. These CVs provide a proportional measure of variability around the mean, allowing comparisons across widely varying values of a variable and between variables. Nineteen different environmental variables and their CVs were initially used to predict the distribution of SR. These were chosen after the collinearity of the potential environmental correlates was tested using the variable inflation factor ensuring that no potential environmental variable had a variable inflation factor >5, using the *vifstep* function in the usdm package (Naimi *et al.*, 2014) in R.

Boosted regression trees (BRT) represent is a machine learning-based method used to model response variables. Unlike traditional statistical methods that rely on null-hypothesis significance testing, BRTs use incremental shrinkage to reduce the contributions of additional variables and control overfitting. BRT models were constructed using the ‘dismo’ library following the methods outlined by Elith *et al.* (2008). The initial models included all variables, and Gaussian BRT models were constructed with specific parameter values. These included a tree complexity of 2, a learning rate of 0.0025 to control the contribution of each tree, a bagging fraction of 0.75 to randomly choose training data to generate each tree, and 10-fold cross-validation to use ten different randomly selected training datasets. Following the initial BRT model construction, models were simplified using procedures outlined by Elith *et al.* (2008). The total explained deviance was calculated as the difference between total and cross-validated deviance divided by the total deviance. The *predict* function was used to predict from the BRT model against the original SR data and to generate a map of predictions. The residuals of the BRT model predictions were compared to elevation ruggedness (as a composite measure of spatial heterogeneity) derived from the SRTM DEM data using the *terrain* function in the raster library (Hijmans, 2022).

## RESULTS

### Spatial heterogeneity vs. resource level

Spatial heterogeneity of environmental variables is a simple metric conceptually and to measure, but it commonly co-varies with mean values as was the case for total N, extractable P, extractable K, NDVI, MAP, and precipitation in the coldest quarter, although there was no significant association of mean PET with its standard deviation (Supplementary Data Fig. S1). pH was the only variable that showed a negative relationship between the mean and standard deviation. This is because pH is the negative log of [H^+^], yielding a positive relationship (*R*^2^ = 0.85, *P* < 0.001) between the mean [H^+^] and the standard deviation of [H^+^] (data not shown). There was a weak (but significant) negative relationship between the CV of extractable P and of PET with their means (within QDSs) and stronger negative relationships for the CVs of soil pH and extractable K and their means ( Fig. S2). By contrast, the CVs of MAP and total N increased with increased mean MAP and total N, whereas there was no relationship between the CVs of NDVI or precipitation in the coldest quarter (i.e. winter rainfall) and their respective means.

### Resources and SR

**Fig. 1.**
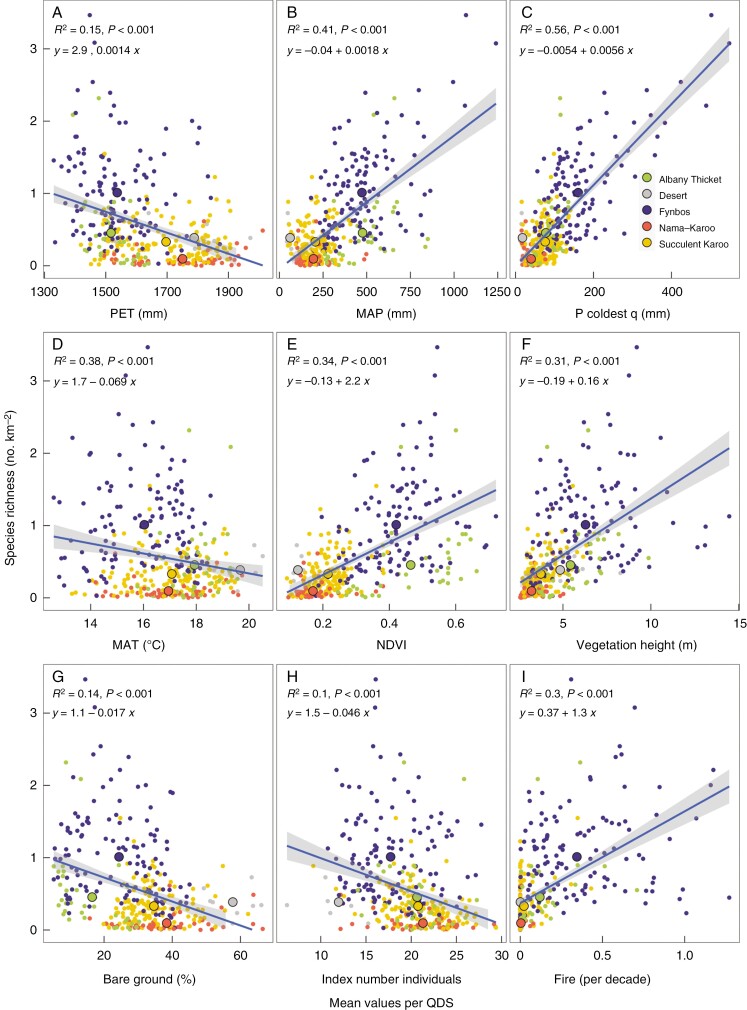
Variation in species richness with (A) mean potential evapotranspiration (PET), (B) mean annual precipitation (MAP), (C) precipitation in the coldest quarter, (D) mean annual temperature (MAT), (E) normalized difference vegetation index (NDVI), (F) vegetation height, (G) the proportion of bare ground, (H) the index of the number of individuals and (I) the fire return interval per quarter degree square (QDS) within the GCFR. The smaller coloured points are the values per quarter degree for the predominant biome in that quarter degree area while the larger points are the means for each biome. The ordinary least square line is shown with a grey band showing the confidence interval and an equation with the *R*^2^ for that line.

We used PET, MAP, precipitation in the coldest quarter, MAT and NDVI (see caveats below) as measures of ‘water–energy’ or non-edaphic resource availability. There was a negative association of SR with PET (Fig. 1), although there was a large degree of variance in SR with PET, particularly at low values of PET. The negative association of PET with SR was particularly attributable to higher SR associated with the Fynbos biome which occupies a region which has low PET relative to other biomes in the region. Both MAP and precipitation in the coldest quarter were strongly positively associated with SR, whereas MAT was negatively associated (Fig. 1).

**Fig. 2.**
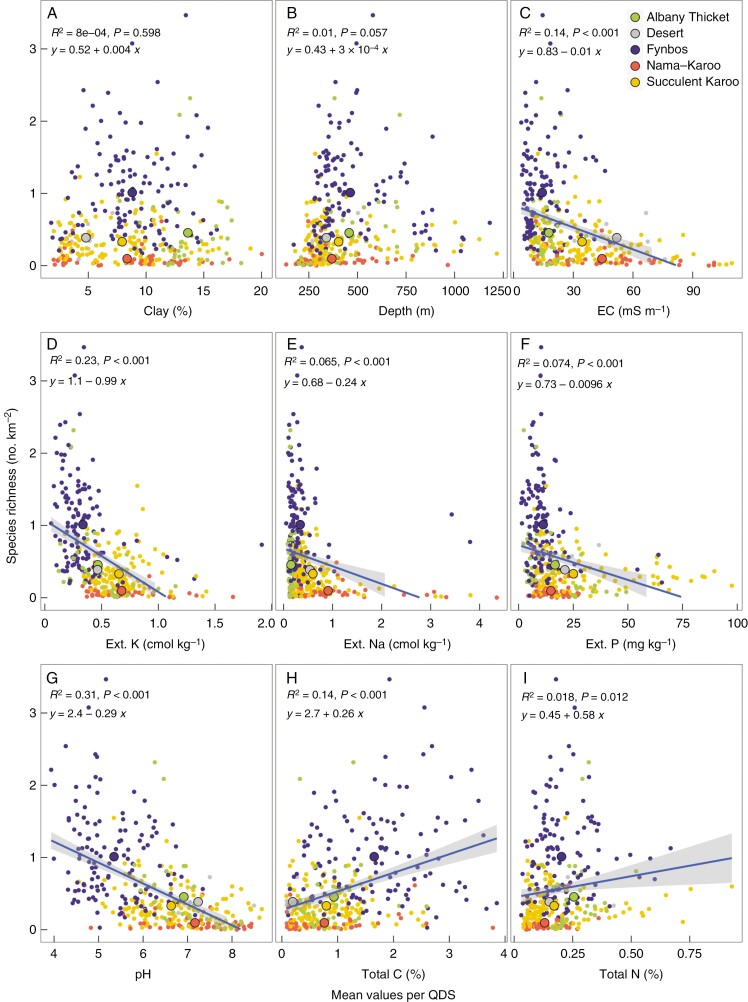
Variation in species richness with the mean values of soil variables that include (A) clay, (B) soil depth, (C) electrical conductivity (EC), (D) extractable K (Ext. K), extractable Na (Ext. Na), (F) extractable P (Ext. P), (G) pH, (H) total C and (I) total N per quarter degree square (QDS) within the GCFR. The smaller coloured points are the values per quarter degree for the predominant biome in that quarter degree area while the larger points are the means for each biome. The ordinary least square line is shown with a grey band showing the confidence interval standard error and an equation with the *R*^2^ for that line where there were significant (*P* < 0.05) correlation coefficients.

The associations between individual mean soil characteristics and SR were variable in directionality (Fig. 2). Whereas the proportion of clay and soil depth were not related to SR, EC, extractable K, Na and P, and pH were all negatively related and total C and N positively related to SR. The negative relationships all featured points from the Fynbos biome at the lower limit of the soil characteristic, but with considerable variability in SR. These negative associations indicate that the highest SR of the region is associated with nutrient-poor soils, but that SR is weakly positively associated with Fynbos biome soils that have higher soil C.

SR was strongly positively related to NDVI (*R*^2^ = 0.34). Despite the fact that NDVI was also highly positively correlated with MAP (*R*^2^ = 0.77, Supplementary Data Fig. S3), SR had a weaker relationship with NDVI than with MAP and precipitation in the coldest quarter.

### Spatial heterogeneity and SR

The CVs of PET, precipitation in the coldest quarter and NDVI were weakly positively correlated with SR (Supplementary Data Fig. S4). The CVs of MAP and MAT were, however, more strongly positively associated with SR ( Fig. S4). The CVs of vegetation height, bare ground and the index of the number of individuals were all positively related to SR and all exhibited a strong contribution of the Fynbos biome to these associations ( Fig. S4). Of these the CV of the index of the number of individuals had the strongest association with SR. The spatial variability in fire within the Fynbos biome was low with greater spatial variability in those biomes that are less fire-prone (cf. Fig. 1). This resulted in a negative association of SR with the spatial variability of fire recurrence. The species-rich Fynbos biome is therefore more spatially heterogenous in water–energy metrics and vegetation characteristics than the other biomes included in the analysis, but less variable with respect to fire.

Unlike the associations between the mean soil characteristics and SR, the CVs of these soil characteristics were all significantly and positively associated with SR (Supplementary Data Fig. S5), suggesting that SR may also be linked to edaphic heterogeneity. The association of SR with the CVs of extractable K and clay were particularly strong. The two outliers (coastal sites) with high extractable Na values had negligible effects on the strength of the regression of SR against the CV of Na. The Fynbos biome had a high degree of spatial heterogeneity in all of the soil properties assessed and was a strong contributor to the association of the CVs with SR.

### Association of SR with bare ground, tree height and a proxy for the number of individuals

NDVI and vegetation height were strongly positively correlated (Supplementary Data Fig. S6, *R*^2^ = 0.56) and vegetation height was also strongly positively related to SR (Fig. 1, *R*^2^ = 0.31). The species-rich Fynbos biome contributed strongly to this relationship. This biome exhibited greater variation in height and SR and also contributed to the positive relationship (*R*^2^ = 0.22) between the CV of vegetation height and SR ( Fig. S4). The percentage of bare ground (i.e. 100 − cover percentage) was negatively associated with SR indicating that areas with less vegetation cover generally have fewer species (Fig. 1). This is consistent with the strong positive correlation (*R*^2^ = 0.41) of both aridity index (MAP/PET) and the CV of aridity index with SR ( Fig. S7).

The association between the index of number of individuals and SR was negative (Fig. 1, *R*^2^ = 0.10). We tested the relationship between NDVI and this index of the number of individuals and found a weak decreasing trend (*R*^2^ = 0.041, *P* < 0.001, Supplementary Data Fig. S8) showing that vegetation with dense foliage (i.e. high NDVI) has fewer individuals.

### Association of SR with temporal heterogeneity

**Fig. 3.**
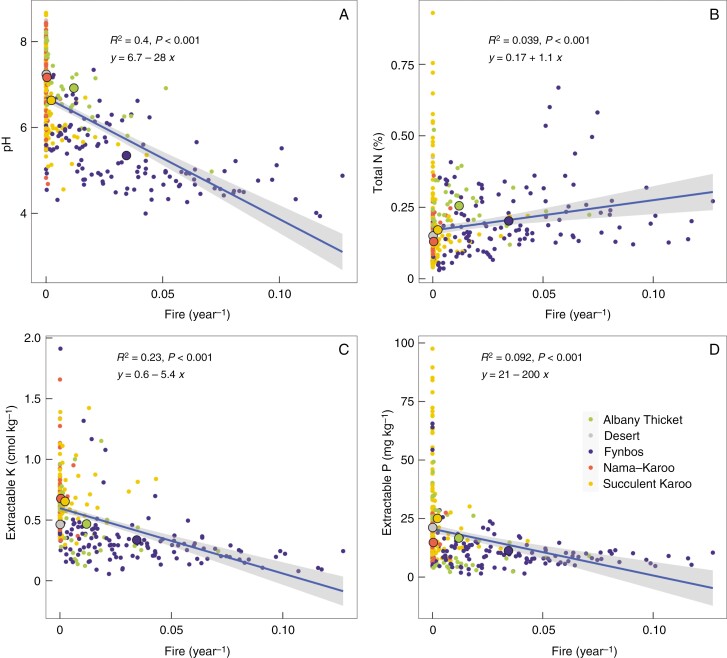
Variation in soil (A) pH, (B) total N, (C) extractable K and (D) P with the fire return interval within the GCFR. The smaller coloured points are the values per quarter degree for the predominant biome in that quarter degree area while the larger points are the means for each biome. The ordinary least square line is shown with a grey band showing the confidence interval and an equation with the *R*^2^ for that line.

There was a positive association of fire frequency with SR that was strongly linked to fires in the Fynbos biome (Fig. 1). Apart from direct effects on vegetation, fire may also alter edaphic properties. Fire frequency had a significant negative relationship with soil pH, and extractable K and P that was largely driven by variability in the fire regime within the Fynbos biome and the fact that arid biomes with more nutrient-rich soils (e.g. Nama Karoo and Succulent Karoo, desert) have few fires (Fig. 3). There was a relatively weak positive association of fire frequency with soil total N, dictated by variability in the Fynbos biome. Apart from temporal heterogeneity induced by fire, increasingly variable seasonal temperatures (i.e. both temperature seasonality and isothermality) were negatively associated with SR (Supplementary Data Fig. S9). By contrast, there was no significant relationship between precipitation seasonality and SR, which differs from the result obtained by Colville *et al.* (2020) who showed a decreasing relationship between SR and ‘seasonality’ measured as the monthly rainfall concentration across a similar region. The discrepancy might be due to the different abundances of points in Colville *et al.* (2020) associated with both the high- and low-seasonality environments.

### Evaluation of predictors of SR

The correspondence between the predictions for each environmental and biotic correlate of SR based on the various hypotheses and the observed outcomes are summarized in Table 1. This shows that there was no support for the energy hypothesis (PET and MAT) and relatively strong support for the role of water (i.e. MAP) as a resource. For edaphic resources the expectation of a humped pattern of SR with soil resources was not met. Only soil C and N exhibited a positive association with SR, the remaining soil properties providing support for nutrient-scarcity driving SR. The MIH was not supported and temporal heterogeneity received only weak and partial support (i.e. fire). The spatial heterogeneity of all variables except that of fire was consistent with the predictions. The contribution of biotic interactions to SR were also supported.

**Table 1.** Comparison of the predicted and observed associations of variables with species richness (SR) for different hypotheses. Orange or yellow indicate negative or non-significant relationships, respectively, relative to the predicted association. For edaphic resources more resources may lead to higher SR, but nutrient scarcity may also drive greater SR. As a consequence these predictions/observations are shaded grey. All variables for the spatial heterogeneity hypothesis are coefficients of variation.

Hypothesis	Variable	Predicted	Observed	Source
Water–energy	PET	+	−	Fig. 1
	MAP	+	+	Fig. 1
	P coldest quarter	+	+	Fig. 1
	MAT	+	−	Fig. 1
	NDVI	+	+	Fig. 1
Edaphic resources	Clay	+/−	0	Fig. 2
	Depth	+/−	0	Fig. 2
	K	+/−	+/−	Fig. 2
	P	+/−	+/−	Fig. 2
	pH	+/−	+/−	Fig. 2
	C	+/−	+/−	Fig. 2
	N	+/−	+/−	Fig. 2
Many individuals hypothesis	Number of individuals	+	−	Fig. 1
	Vegetation height	−	+	Fig. 1
Temporal heterogeneity	Seasonality MAP	+	0	Fig. S9
	Seasonality MAT	+	−	Fig. S9
	Fire	+	+	Fig. 1
Spatial heterogeneity	PET	+	+	Fig. S4
	MAP	+	+	Fig. S4
	P coldest quarter	+	+	Fig. S4
	MAT	+	+	Fig. S4
	NDVI	+	+	Fig. S4
	Vegetation height	+	+	Fig. S4
	Bare ground	+	+	Fig. S4
	Number of individuals	+	+	Fig. S4
	Fire	+	−	Fig. S4
	Clay	+	+	Fig. S4
	Depth	+	+	Fig. S5
	K	+	+	Fig. S5
	P	+	+	Fig. S5
	pH	+	+	Fig. S5
	Clay	+	+	Fig. S5
	N	+	+	Fig. S5
Biotic interactions	NDVI	+	+	Fig. 1
	Vegetation height	+	+	Fig. 1

MAP, mean annual precipitation; MAT, mean annual temperature; NDVI, normalized difference vegetation index; PET, potential evapotranspiration.

**Fig. 4.**
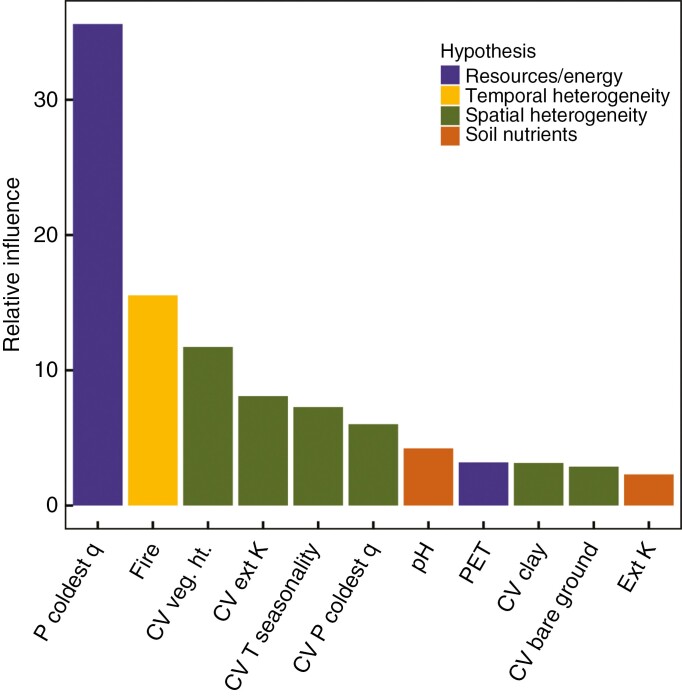
The relative influence of variables retained in a simplified boosted regression tree model of GCFR species richness. The retained variables included precipitation in the coldest quarter (P coldest q), fire frequency (Fire), soil pH, potential evapotranspiration (PET), extractable K (Ext K) and the CVs (standard deviation/mean) of vegetation height (Veg. Ht.), soil extractable K (ext K), temperature seasonality (T seasonality) precipitation in the coldest quarter (P coldest q), clay and bare ground. The bars are coloured to indicate broad hypotheses into which the variables may be grouped, although some may fit with more than one category (e.g. soil nutrients could be included with resources/energy).

**Fig. 5.**
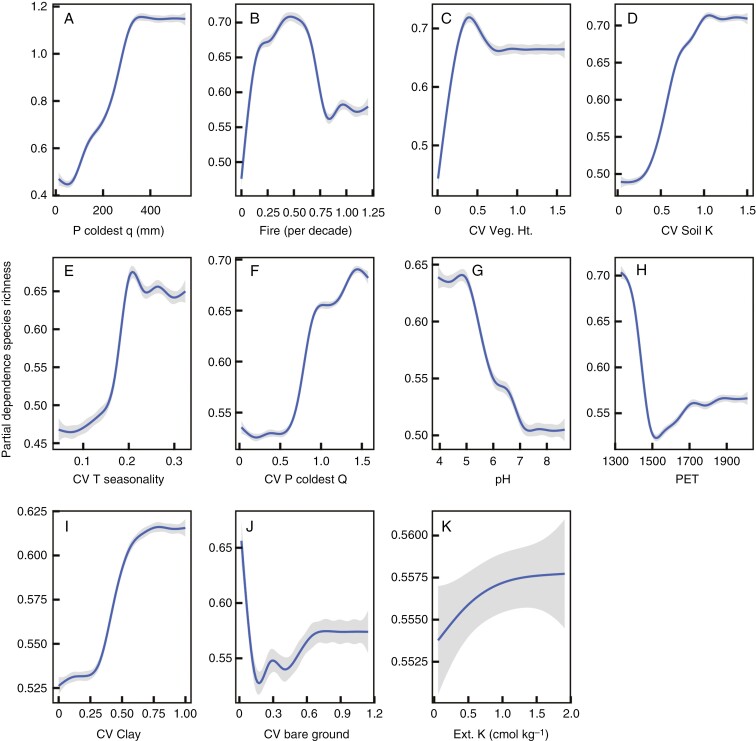
Partial dependence plots produced from a boosted regression tree model for each of the variables detailed in the relative influence plot (Fig. 4). The blue lines are fitted to the predicted data using a generalized additive model, and the grey band shows the confidence interval of the partial dependence. The plots are arranged in order of decreasing relative influence.

The final simplified boosted regression tree model retained 11 predictors with a residual deviance of 0.065 out of a total deviance of 0.319 and a cross-validation correlation of 0.79 ± 0.02 (Fig. 4). Precipitation in the coldest quarter was by far the strongest predictor (34 % of the variance) of SR, which together with PET (a negative predictor, see Fig. 5) indicates a strong dependence of SR on water availability. Although fire frequency is related to temporal heterogeneity it could also create spatial heterogeneity, but the spatial CV of fire was not retained in the simplified BRT model. Vegetation height and bare ground are biotic variables, the CVs of which were retained in the model explaining SR. The only soil variables that were retained in the model were pH and extractable K, although the variances of extractable K and clay in the soil were also retained. The CV of several variables (six out of 11 variables) were retained in the model, suggesting a strong degree of dependence on spatial heterogeneity induced by climatic, edaphic and biotic variability.

**Fig. 6.**
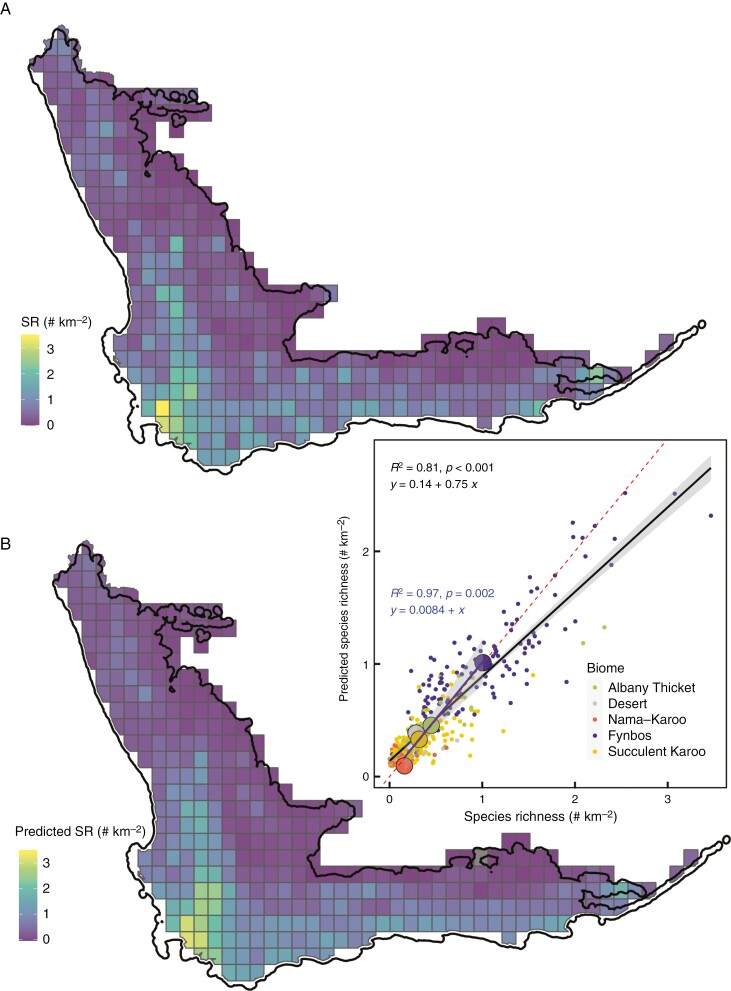
Predicted species richness (lower map, B) for each quarter degree (QDS) within the GCFR vs. the observed species richness (upper map, A). The black border shows the coastline and the inland border of the GCFR. (C) Comparison between observed and predicted SR in which the smaller coloured points are the values per quarter degree for the predominant biome in that quarter degree area while the larger points are the means for each biome. The black line is a linear fit to the QDS data and the blue line represents the fit for the biome averages. The ordinary least square lines are shown with a grey band showing the confidence interval and an equation with the *R*^2^ and *P*-value for each line. The broken red line is the 1:1 line.

Partial dependence plots (Fig. 5) broadly correspond to the relationships between the bivariate plots (e.g. Figs 1 and 2) but are modelled responses with all other variables held at their average values. The common pattern for all except pH, PET and the CV of bare ground was for SR to increase with the increase in the predictor variables, before reaching a point beyond which further increases in SR did not occur. Fire return frequency was also an exception to the pattern in that fire return frequencies of >0.47 fires per decade were associated with decreasing SR. The prediction of SR was well correlated with observed SR (*R*^2^ = 0.81, Fig. 6), but the model under-predicted the higher SR. This is also evident from the distribution map of predicted SR compared to the actual SR per QDS (Fig. 6).

### Putative SR feed-forward effect

**Fig. 7.**
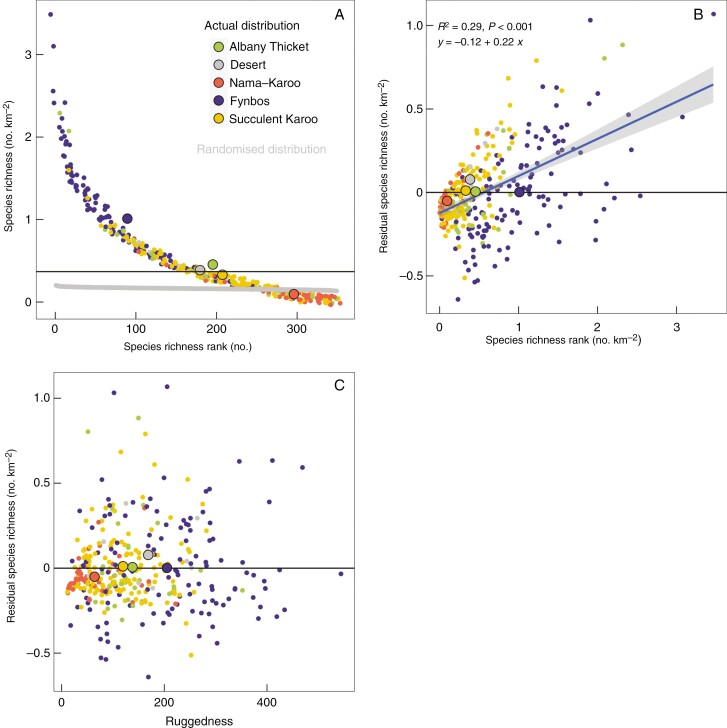
The species richness rank curves (SRR) points showing (A) the variation in SR with the rank of species richness within the GCFR. The smaller coloured points are the values per quarter degree for the predominant biome in that quarter degree area while the larger points are the means for each biome. Note that the points are jittered along the *x*- and *y*-axes to reduce overlap of the points. The same data were randomized (each species randomly reassigned to a QDS) to show the expected ‘normal’ distribution (grey line). The median species richness for all QDSs is shown by the horizontal black line. (B) Residuals of the boosted regression tree model plotted vs. observed species richness and (C) vs. topographic heterogeneity (ruggedness). For the residuals vs. species richness an ordinary least square linear regression line is shown with a grey band showing the confidence interval and an equation with the *R*^2^ and *P*-value for that line.

There is no direct way to assess the extent to which SR exerts a positive reinforcing feedback on itself in these data. One indirect approach is to determine the shape of the species richness distribution (SRR, Fig. 7) which follows a hollow shape and differs from a randomized distribution (created by randomly assigning each species to a QDS in the region). The median SR was 0.37 species km^−2^ whereas the mean was 0.56 species km^−2^. This shows that most QDSs (64 %) have an SR that is below the average. This is driven by the fact that a few QDSs have very high SR, following a power relationship that could be due to feed-forward effects combined with regional hotspots of SR (e.g. associated with spatial heterogeneity). This high SR was mostly associated with the Fynbos biome, although some Albany thicket and succulent Karoo QDSs also contributed.

The residuals of the BRT model showing the unexplained variance of SR were positively associated with SR (Fig. 7) with a tendency to under-estimate the higher SR sites. This probably indicates that there were additional important predictors of SR that were omitted from the model. These might include biotic factors such as a feed-forward effect contributing to SR, particularly in the more species-rich sites. The residuals of the BRT model were not related to topographic ruggedness (Fig. 7), which has been widely used as a proxy for environmental heterogeneity within South Africa (Thuiller *et al.*, 2006) showing that the BRT model accounted for the contribution of environmental heterogeneity to SR across all varying SR values. The covariances of SR with both NDVI and vegetation height (Fig. 1) might also be interpreted as being due to biotic feedbacks due to the possible differentiation of plant niches in more densely vegetated areas in which access to light is limited by vegetation stature. However, other omitted explanatory variables might also contribute to the unexplained variance. For example, regions of high climatic stability through the Cenozoic (Colville *et al.*, 2020) could also explain the larger residuals with higher SR.

## DISCUSSION

PET, which increases with light, temperature and wind and decreases with relative humidity, has been a useful measure of ‘energy’ for ecosystems where light and temperature are limitations on plant growth (e.g. Kreft and Jetz, 2007). Within South Africa (Hoffman *et al.*, 1994) and the GCFR (Fig. 1), however, this measure of energy availability was negatively associated with SR. This is probably linked to the fact that, regionally, as PET increases so does aridity leading to decreases in plant canopy cover (Supplementary Data Fig. S7), raising the question as to whether this measure of ‘energy’ is actually relevant in temperate zones (Hawkins *et al.*, 2003). Indeed, the minimum PET within the GCFR (1330 mm year^−1^) is toward the higher end of the positive relationship between PET and global vascular plant SR reported by Kreft and Jetz (2007). By contrast, the positive relationships between both increased rainfall (MAP and precipitation in the coldest quarter) and NDVI with SR suggest that these metrics of resource availability in the environment matter more for determining SR (Table 1). Since MAP in the GCFR is relatively low (average = 324 mm year^–1^, *n* = 350 QDS) it is perhaps not surprising that SR is positively associated with water inputs, instead of the input of ‘energy’ as measured by PET that is accompanied by the loss of water.

A fundamental component of ‘OCBIL theory’ is the association of SR with nutrient-poor and highly leached soils (Hopper *et al.*, 2021). This association is evident from the negative relationship of SR with soil EC, K, Na, P and pH within the GCFR (Fig. 2). Species coexistence might be favoured by differential sensitivities of different species to nutrient deficiency. The association of Proteaceae SR with low-P oligotrophic environments (Hayes *et al.*, 2021) suggests that there would be a particularly strong negative association between SR and P, especially considering the many adaptations of regional plants to low-P soils (reviewed by Cramer *et al.*, 2014). There is also weaker inter-specific competition for P than for other nutrients (e.g. N) due to its low mobility in soil, possibly contributing to greater coexistence and higher SR with scarce P (Güsewell, 2004). The weaker negative association of SR with P relative to other edaphic properties (e.g. EC, K, pH) may mean that P is less important for SR than has been suggested, or point to deficiencies in either the SR or soil data. A deficiency of the soil data used in this study is the lack of total P, which is probably more important for native species than available P. Available P is an agricultural measure that may be less relevant to native species that have adaptations to acquire scarce P (e.g. Lambers *et al.*, 2010) and is much more temporally variable than total P. Both soil total N and C, however, increased with increasing SR, possibly because denser (i.e. lower bare ground cover) and taller vegetation was associated with greater SR (Fig. 1). Apart from N deposition, most soil N is biotically fixed by symbiotic and free-living microorganisms, and this probably reflects the vegetation characteristics (reviewed by Cramer *et al.*, 2014). A feedback exists, however, in which more N favours more vegetation, and more vegetation favours greater N and C retention in the ecosystem through the formation of organic material in the soil (Townsend *et al.*, 1995). For example, in Afromontane forests in the GCFR, nutrients accumulate in soils that are derived from nutrient-depauperate geologies (Cramer and Verboom, 2017). Nevertheless, fires, which are reliant on fuel-loads (Kraaij and van Wilgen, 2014), also volatilize N (Stock and Lewis 1986), potentially limiting the strength of the association of biomass and SR with soil N. The negative associations of several soil nutrients with SR was strongly determined by a pattern of large variances in SR values at low values of the edaphic properties, resulting in triangular distributions of points in the plots (Fig. 2). This raises the question as to whether the SR–nutrient association is causal, since many areas with low SR also have low edaphic values. The exception to this pattern was pH which had a stronger negative association with SR than other nutrients, and is a powerful predictor of overall nutrient availability due to its association with leaching (Cramer and Hoffman, 2015).

For understanding of SR patterns, however, a more integrated measure of resources available to plants may be NDVI, since this must reflect the ability of the total environment (including edaphic factors) to support plant growth. The use of NDVI is, however, subject to the limitation that herbivory and fire may remove part of the standing biomass, thus limiting NDVI and possibly explaining why NDVI was a weaker univariate predictor of SR than MAP. NDVI might also be considered a ‘biotic’ variable in which variability in NDVI contributes to niche diversity (Power *et al.*, 2017). Despite these caveats, the positive association of NDVI with SR may suggest that greater productivity within the GCFR is associated with higher SR, which conflicts with the notion that nutrient-scarcity per se is a driver of SR.

Since spatial variability in environmental attributes is highly and mostly positively correlated with the levels of those attributes (e.g. Supplementary Data Fig. S1) it is difficult to unscramble whether SR is associated with the resource level per se, or its variability. To this end we have used the CV of each variable to indicate the variability because this controls for the level of a particular resource. The variability that is relevant for determining SR is that which creates niche diversity (Stein *et al.*, 2014). This may be better associated with the spatial variability measured here with the standard deviation of environmental variables. For example, for extractable K it may be that the standard deviation within a QDS is more important for separating niches than the CV. The CV may be high for low values of extractable K, but are niches differentiated by small variations in small values (possibly yielding high CVs) or by the total variability (i.e. standard deviation)? One might argue that the CV is a better metric because it takes into account variability, even at low levels of extractable soil K. Each variable could be debated in this manner, but the strong relationship between most environmental variables and their standard deviations means that whenever the mean of an attribute is retained in a model predicting SR, this should be interpreted as indicating not only that the level of that attribute may contribute, but also possibly its variability. Indeed, this is a general comment about associations of environmental variables with SR in the literature (e.g. Thuiller *et al.*, 2006; Kreft and Jetz, 2007). The negative association of nutrients with SR might also reflect an association with the spatial variability of the edaphic properties. Supporting this, the CV of the edaphic properties examined were all positively related to SR, and the relationships between the CVs of K and P with SR were stronger than the relationships between mean K or P with SR. This indicates that it may be the heterogeneity of these edaphic properties, rather than their low values per se, that explains SR.

The MIH is somewhat allied to the water–energy relationship in that more ‘energy’ across latitudinal gradients has been suggested to result in more individuals and thus more species (reviewed by Storch *et al.*, 2018). By contrast, within the GCFR there was a negative relationship between the index of the number of individuals and SR (Table 1). This together with the negative association of bare ground and the positive association of vegetation height with SR could be a consequence of relatively species-poor QDSs being in more arid-zone biomes (e.g. Nama-Karoo and Succulent Karoo) with many smaller stature plants and more bare ground. These arid zones are also less topographically and environmentally heterogenous, also possibly contributing to fewer species. The negative association of SR with the index of the number of individuals is consistent with the positive association of vegetation height and SR, bearing in mind the caveat that alien invasion could also partially contribute to vegetation heights, although regionally alien invasion reduces indigenous SR (Mostert *et al.*, 2017). Whether the association between SR and vegetation height is potentially causal is debatable since vegetation height is also strongly corelated with rainfall and NDVI. Nevertheless, the CV of vegetation height was associated with SR, possibly because this indicates a diversity of vegetation types within a region that might include open- and closed-canopy vegetation, therefore reflecting species turnover. Therefore, there is no evidence that the generally small sizes of GCFR plants and the fact that there are many individuals predict SR in this region.

The species-rich Fynbos biome was strongly associated with higher fire frequencies (mean ± s.e., 0.35 ± 0.03 fires per decade, Fig. 1) and more regular fires than the other GCFR biomes (0.03 ± 0.005 fires per decade). Although the current fire frequency in some areas has been altered by humans, resulting in both more-frequent (human-induced ignitions) and less-frequent fires (areas protected from fires; Kraaij and van Wilgen, 2014), vegetation does not carry fire unless there is sufficient fuel load (van Wilgen *et al.*, 1990). The positive association between fire and SR may be due to fire acting as a periodic spatially variable reset of the ecosystem allowing temporal species turnover following fire (e.g. Cocks and Stock, 2001; Power *et al.*, 2010). Apart from the direct effects of fire on SR, fire may also drive nutrient loss from burnt areas and enrich neighbouring less-flammable vegetation (Cramer *et al.*, 2019). Indeed, in this analysis higher fire frequencies were particularly associated with lower pH, which commonly indicates greater leaching and nutrient impoverishment. The weak positive association of total N with fire frequency may be because total N is associated with NDVI (*R*^2^ = 0.18, *P* < 0.001, data not shown). Fire is a complex abiotic variable varying in frequency and intensity that is partially determined by vegetation characteristics and the consequences for SR are correspondingly complicated. For example, Richter *et al* (2019) found a hump-shaped relationship between plant diversity and fire severity. The direction of causality between fire and nutrients is also not entirely obvious since, while fire does release nutrients (e.g. Stock and Lewis, 1986), it may also be the case that medium-stature (in the context of the GCFR), open-canopy, nutrient-limited Fynbos is more prone to fires (e.g. fires do not commonly burn into Afromontane forests adjacent to Fynbos; Kraaij and van Wilgen, 2014), or a feed-back between these two.

While many hypotheses have been put forward to explain the uniquely high SR of the GCFR, it has not been possible to quantitatively evaluate the contributions of those individual hypotheses to SR. This remains problematic since, as is clear from the foregoing discussion, association does not necessarily imply causation. For example, while precipitation in the coldest quarter is strongly related to SR (Fig. 1), is that because winter rainfall favours species accumulation or just that areas with higher SR happen to be in winter rainfall climates? Nevertheless, the BRT model at least reveals the most likely candidate predictors of SR. From this, SR was associated with larger amounts of winter rainfall >300 mm year^–1^ and PET < 1500 mm year^–1^ within the GCFR, linking higher SR to water availability. Fire frequency was also a strong predictor of SR, no doubt for diverse reasons that include spatial and temporal heterogeneity and possibly ecophysiological specializations to fire. By contrast, the direct effects of soil edaphic properties on SR were rather minor predictors of SR. This is consistent with what we reported previously for South Africa as a whole and something that we found surprising since there is a wealth of information that links β-diversity to edaphic turnover (Cramer and Verboom, 2017). Nevertheless, spatial variability in edaphic properties is strongly linked to the value of edaphic characteristics (Supplementary Data Fig. S1) and the CVs of K and clay were both retained in the model. Furthermore, when vegetation height, which might also partially reflect nutrient availability (e.g. Cramer, 2012) was dropped from the BRT model, both soil extractable P and its CV were also retained in the model, without penalizing predictive power (data not shown; *R*^2^ = 0.82, *P* < 0.001). While there are many caveats about the interpretation of these multiple associations with SR, the fact is that over the 350 QDSs within and bordering the GCFR we were able to predict SR with 81 % accuracy.

The contributions of biotic interactions to SR may be greatest in the tropics (Schemske *et al.*, 2009), but the positive feedback effect of biotic interactions on SR is conceivably a general feature of ecological systems. Apart from studies of such interactions on an individual basis (e.g. the fraction of animal-dispersed plants, symbiotic ant–plant mutualisms, allocation to structural defences against herbivores; Schemske *et al.*, 2009) there is little evidence for the importance for the SR of such interactions in real ecosystems. The hollow shape of the SRR may provide evidence for a feed-forward effect of SR on SR, although the concentration of species in particular QDSs could also be due to environmental heterogeneity and the curve could also simply reflect the concentration of species in a few QDSs. Our BRT model included multiple measures of heterogeneity and thus may be interpreted as having included this heterogeneity in SR predictions. The model residuals are due to unexplained variances that may be associated with any contribution to SR that was omitted from the models. The increasing magnitude of the model residuals with SR could be ascribed to biotic feed-forward effects. The fact that the residuals were not associated with variations in topographic heterogeneity lends support to this claim since topographic heterogeneity was excluded from the model, but has been used previously as a proxy for environmental heterogeneity (Thuiller *et al.*, 2006) since it captures spatial variability in many environmental characteristics including temperature, precipitation and edaphic properties (Cramer and Verboom, 2017). This unexplained variability in the model accounted for only 19 % of the total variability over the full set of QDSs, although the maximum residual SR was 1.1 species km^−2^ amounting to 31 % of the SR of that particular QDS, which is located in the Kogelberg Centre, an area renowned for SR and endemicity (van Mazijk *et al.*, 2021). It is equally possible, however, that historical factors (e.g. biome stability; Colville *et al.*, 2020) that were not included in this study also contributed to the unexplained variance. The putative role of biotic feedbacks thus remains largely speculative.

## CONCLUSION

The SR of the GCFR is associated with a plethora of different factors, many of which we were not able to assess in this analysis (e.g. role of refugia, historical balance between speciation and extinction rates). Nevertheless, we conclude that the high SR areas (i.e. QDSs) of the region are associated with more mesic sites with a moderate availability of rainfall in winter (>300 mm), regular but not frequent fires (optimum = 0.47 fires per decade), and spatial heterogeneity in climatic and edaphic variables. Biotic feed-forward effects may also contribute to SR although there is uncertainty about this due to a lack of direct evidence. Surprisingly, soil nutrient impoverishment had a relatively weak relationship with SR and the MIH was not supported for SR in the region. These patterns of SR may be disrupted in surprising ways by ongoing climate change (e.g. Parmesan and Hanley, 2015).

## SUPPLEMENTARY DATA

Supplementary data are available at *Annals of Botany* online and consist of the following.

Figure S1. The variation in the standard deviations of soil total N, extractable P, extractable K, pH, normalized difference vegetation index values, mean annual precipitation, precipitation in the coldest quarter and potential evapotranspiration with their respective mean values per quarter degree within the GCFR. Figure S2. The variation in coefficients of variation of soil total N, extractable P, extractable K, pH, normalized difference vegetation index values, mean annual precipitation, precipitation in the coldest quarter and potential evapotranspiration with their respective mean values per quarter degree within the GCFR. Figure S3. Variation in NDVI with potential mean annual precipitation within the GCFR. Figure S4. Variation in species richness with the coefficient of variation of potential evapotranspiration, mean annual precipitation, precipitation in the coldest quarter, mean annual temperature, normalized difference vegetation index, vegetation height, the proportion of bare ground, the index of the number of individuals and the fire return interval per quarter degree square within the GCFR. Figure S5. Variation in SR with the coefficient of variation of soil variables that include clay, soil depth, electrical conductivity, extractable K, extractable Na, extractable P, pH, total C and total N within the GCFR. Figure S6. Variation in vegetation height with normalized difference vegetation index within the GCFR. Figure S7. Variation in species richness with aridity index and the CV of the aridity index per quarter degree square within the GCFR. Figure S8. Variation in the index of the number of individuals with normalized difference vegetation index within the GCFR. Figure S9. Variation in species richness with temperature seasonality, isothermality and precipitation seasonality per quarter degree square within the GCFR.

mcad134_suppl_Supplementary_Material
