# Supplementary material for: Quantitative evaluation of the drivers of species richness in a Mediterranean ecosystem (Cape, South Africa)
Source: Ann Bot. 2023 Sep 15;133(5-6):801–18. doi: 10.1093/aob/mcad134 (PMC11082525; doi:10.1093/aob/mcad134)
Supplement: mcad134_suppl_Supplementary_Material [file mcad134_suppl_supplementary_material.docx]

**Supplementary Material**


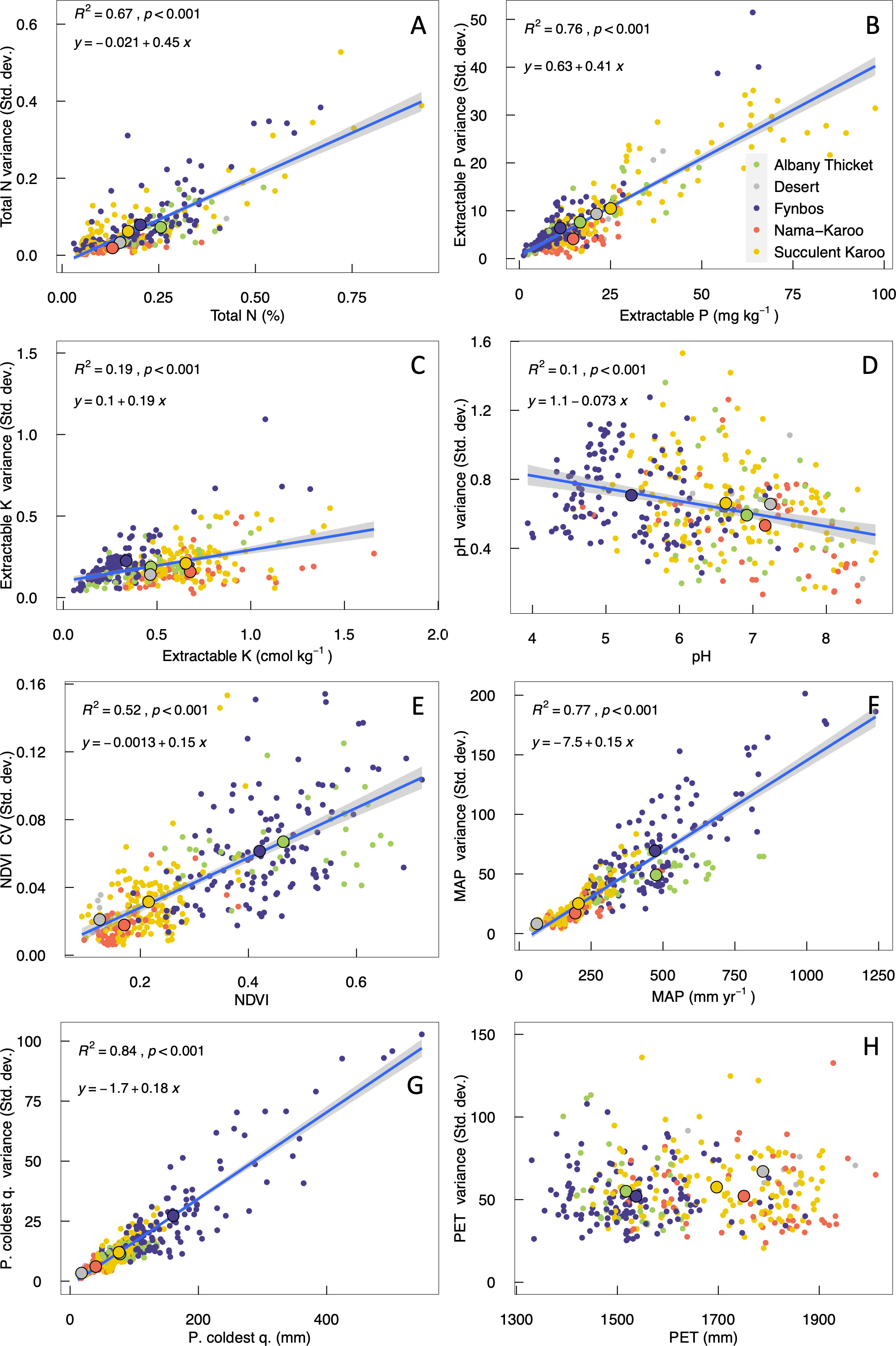


Figure S1. The variation in the standard deviations of A) soil Total N, B) Extractable P, C) Extractable K, D) pH, E) normalised difference vegetation index (NDVI) values, F) mean annual precipitation (MAP), G) precipitation in the coldest quarter (P coldest quarter) and H) potential evapotranspiration (PET) with their respective mean values per quarter degree within the GCFR. The smaller coloured points are the values per quarter degree for the predominant biome in that quarter degree area while the larger points are the means for each biome. The ordinary least square line is shown with a grey band showing the confidence interval and an equation with the R^2^ for that line where significant (P < 0.05).


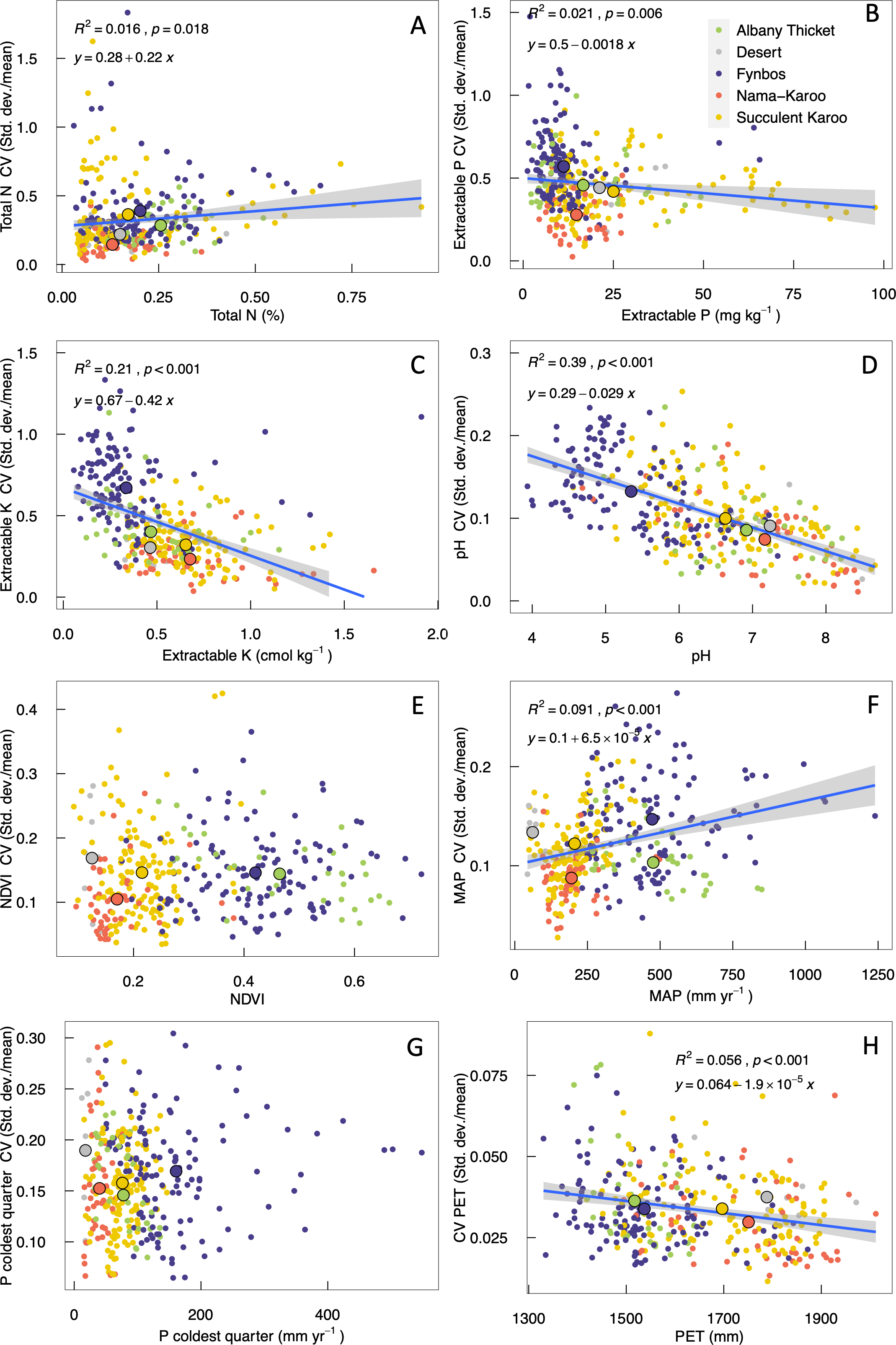


Figure S2. The variation in coefficients of variation (CV, standard deviation/mean) of A) soil Total N, B) Extractable P, C) Extractable K, D) pH, E) normalised difference vegetation index (NDVI) values, F) mean annual precipitation (MAP), G) precipitation in the coldest quarter (P coldest quarter) and H) potential evapotranspiration (PET) with their respective mean values per quarter degree within the GCFR. The smaller coloured points are the values per quarter degree for the predominant biome in that quarter degree area while the larger points are the means for each biome. The ordinary least square line is shown with a grey band showing the confidence interval and an equation with the R^2^ for that line where significant (P < 0.05).


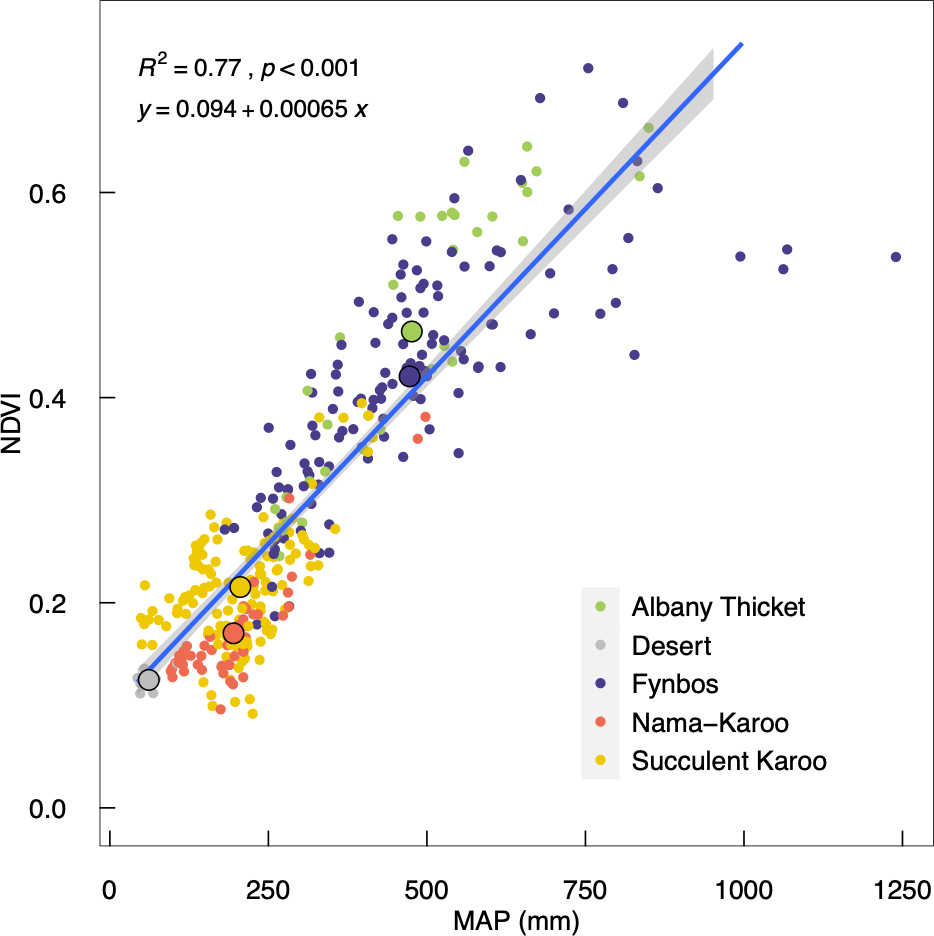


Figure S3. Variation in NDVI with potential mean annual precipitation (MAP) within the GCFR. The smaller coloured points are the values per quarter degree for the predominant biome in that quarter degree area while the larger points are the means for each biome. The ordinary least square line is shown with a grey band showing the confidence interval and an equation with the R^2^ for that line.


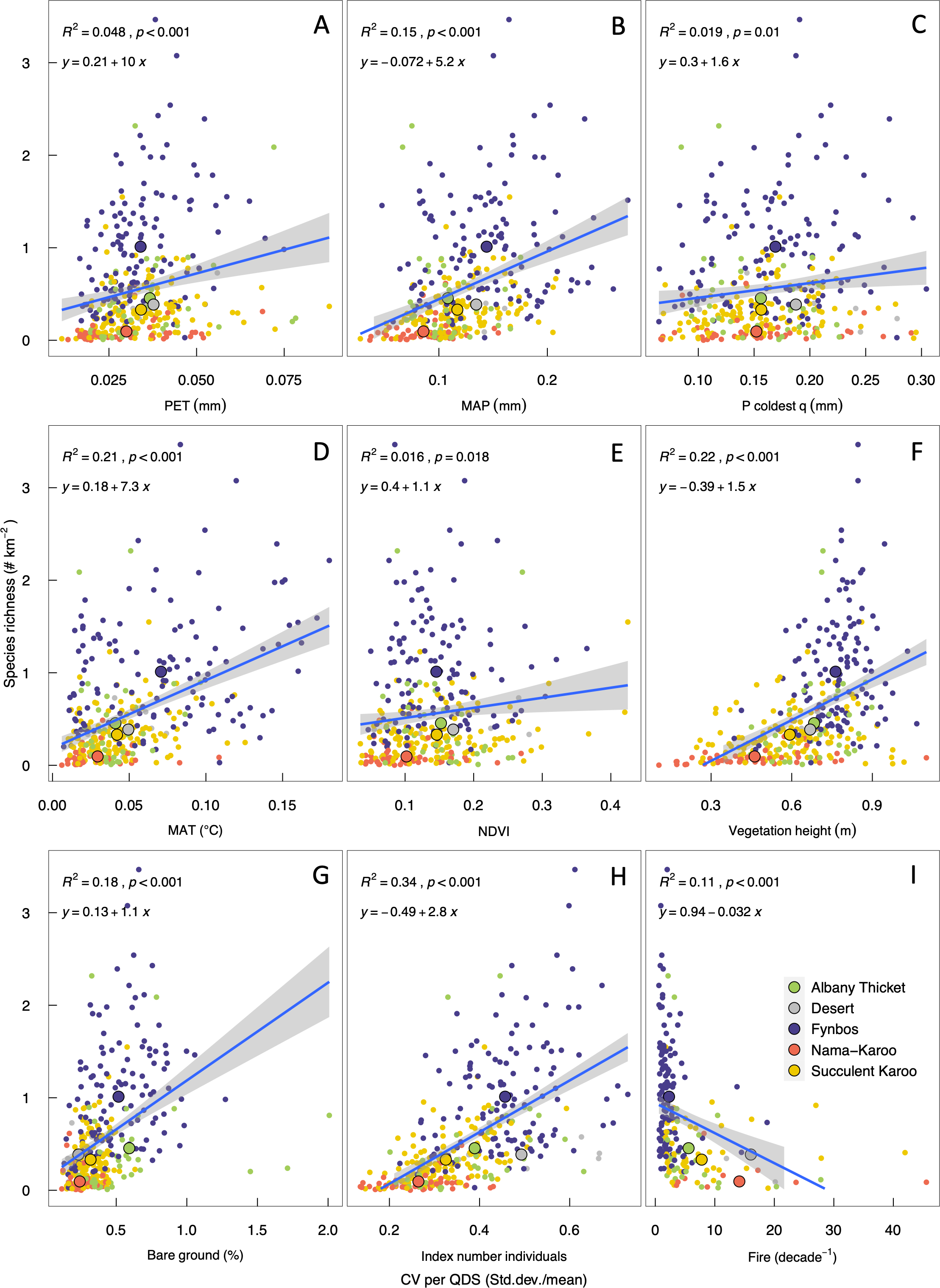


Figure S4. Variation in species richness with the coefficient of variation (CV, standard deviation/mean) of A) potential evapotranspiration (PET), B) mean annual precipitation (MAP), C) precipitation in the coldest quarter, D) mean annual temperature (MAT), E) normalised difference vegetation index (NDVI), F) vegetation height, G) the proportion of bare ground, H) the index of the number of individuals and I) the fire return interval per quarter degree square within the GCFR. The smaller coloured points are the values per quarter degree for the predominant biome in that quarter degree area while the larger points are the means for each biome. The ordinary least square line is shown with a grey band showing the confidence interval and an equation with the R^2^ for that line.


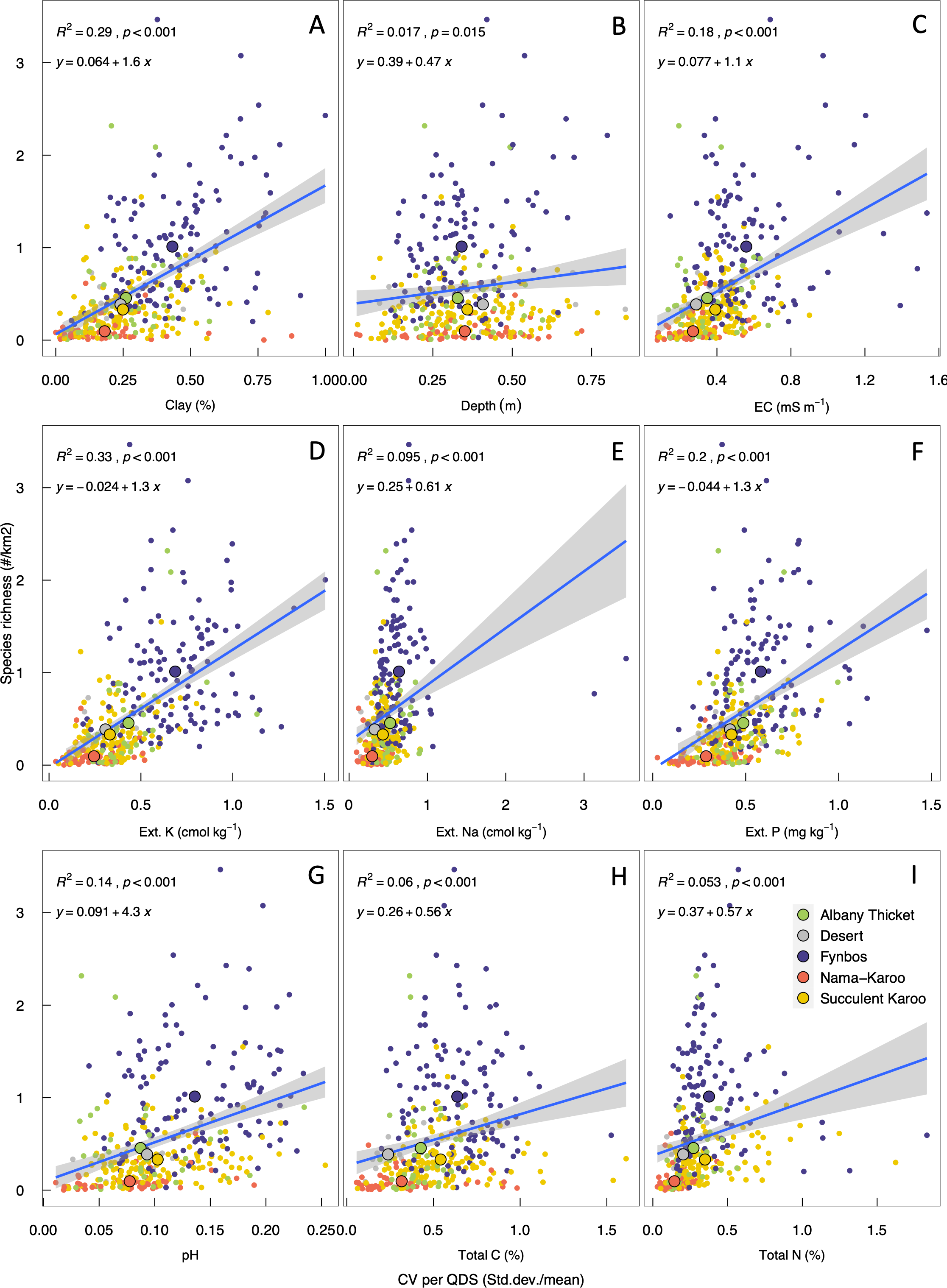


Figure S5. Variation in SR with the coefficient of variation (CV, standard deviation/mean) of soil variables that include A) clay, B) soil depth, C) electrical conductivity (EC), D) extractable K (Ext. K), E) extractable Na (Ext. Na), F) extractable P (Ext. P), G) pH, H) Total C and I) Total N within the GCFR. The smaller coloured points are the values per quarter degree for the predominant biome in that quarter degree area while the larger points are the means for each biome. The ordinary least square line is shown with a grey band showing the confidence interval and an equation with the R^2^ for that line.


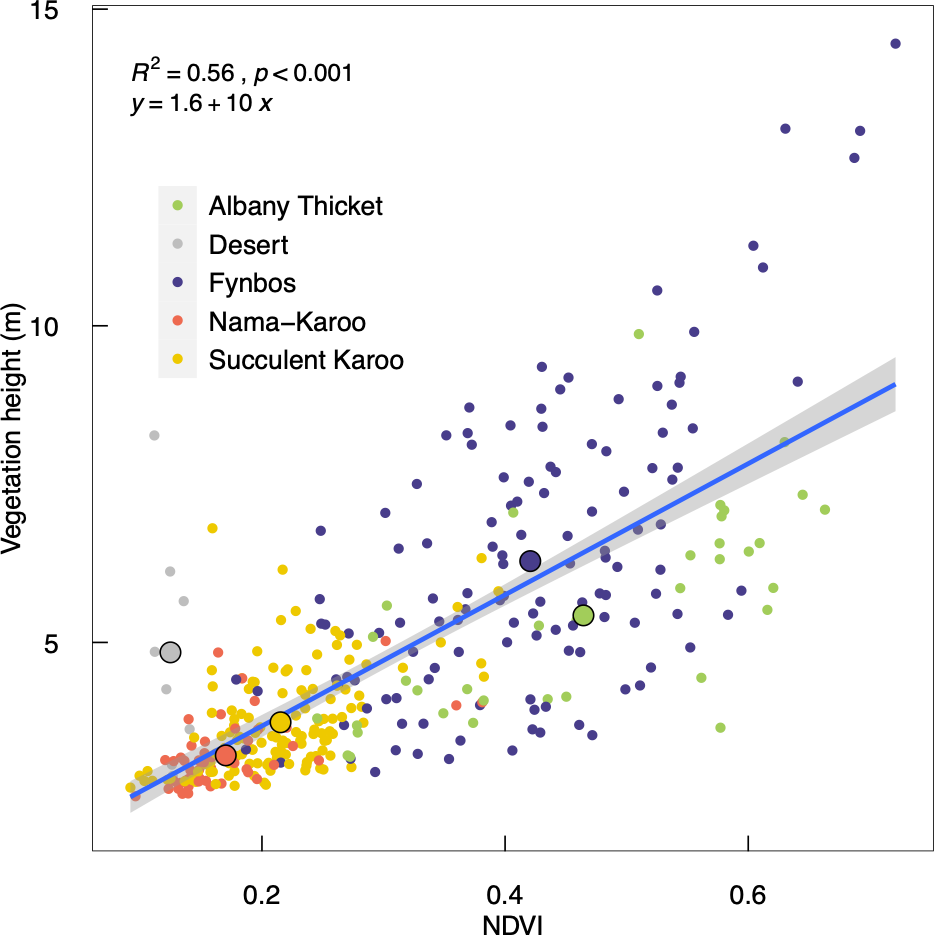


Figure S6. Variation in vegetation height with normalised difference vegetation index (NDVI) within the GCFR. The smaller coloured points are the values per quarter degree for the predominant biome in that quarter degree area while the larger points are the means for each biome. The ordinary least square line is shown with a grey band showing the confidence interval and an equation with the R^2^ for that line.


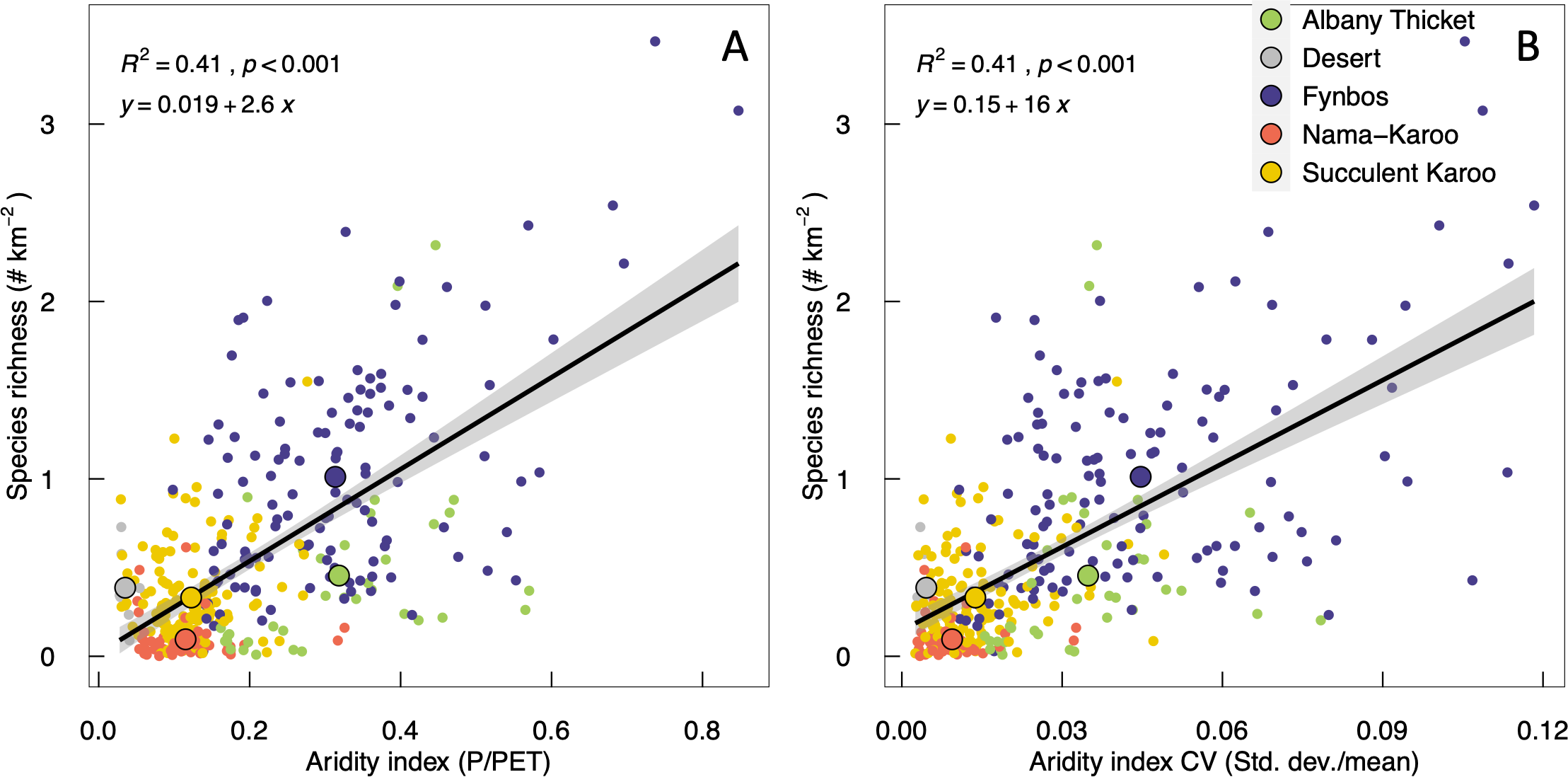


Figure S7. Variation in species richness with A) aridity index (mean annual precipitation/potential evapotranspiration, P/PET) and B) the CV of the aridity index (standard deviation/mean) per quarter degree square within the GCFR. The smaller coloured points are the values per quarter degree for the predominant biome in that quarter degree area while the larger points are the means for each biome. The ordinary least square line is shown with a grey band showing the confidence interval and an equation with the R^2^ for that line.


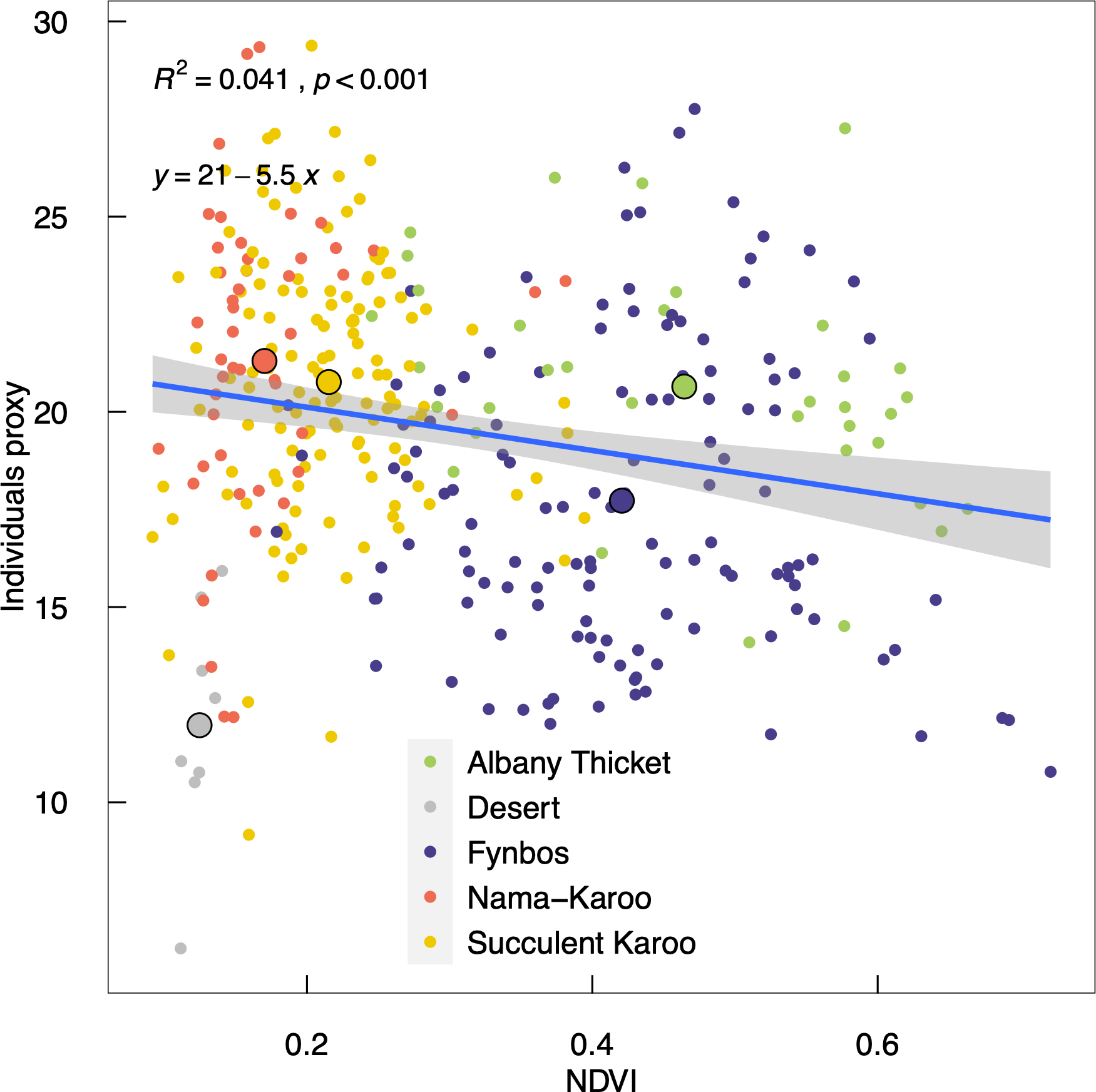


Figure S8. Variation in the index of the number of individuals with normalised difference vegetation index (NDVI) within the GCFR. The smaller coloured points are the values per quarter degree for the predominant biome in that quarter degree area while the larger points are the means for each biome. The ordinary least square line is shown with a grey band showing the confidence interval and an equation with the R^2^ for that line.


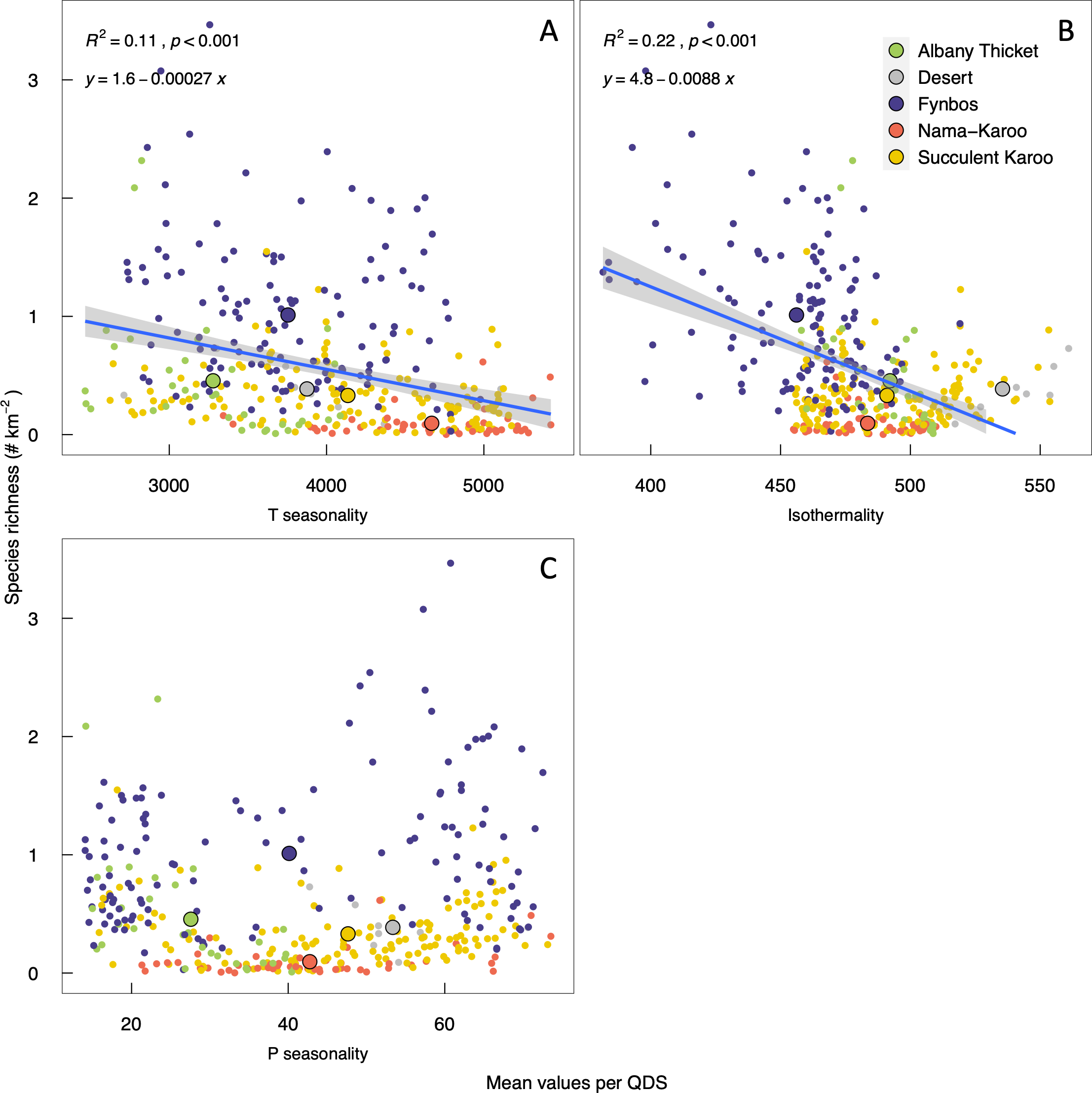


Figure S9. Variation in species richness with A) temperature seasonality (standard deviation of mean monthly temperatures), B) isothermality (mean monthly temperature range divided by minimum temperature) and C) precipitation seasonality (standard deviation of mean monthly precipitation) per quarter degree square within the GCFR. The smaller coloured points are the values per quarter degree for the predominant biome in that quarter degree area while the larger points are the means for each biome. The ordinary least square line is shown with a grey band showing the confidence interval and an equation with the R^2^ for that line where significant (P < 0.05).
